# Supplementary material for: Population-wide analysis of differences in disease progression patterns in men and women
Source: Nat Commun. 2019 Feb 8;10:666. doi: 10.1038/s41467-019-08475-9 (PMC6368599; doi:10.1038/s41467-019-08475-9)
Supplement: Supplementary file 1 — Supplementary Information [file 41467_2019_8475_MOESM1_ESM.docx]

Supplementary Information to:

­

Population-wide analysis of differences in disease progression patterns in men and women

Westergaard et al.

# Supplementary Note 1

## Global Burden of Disease

### Global Burden of Disease Categories

The Global Burden of Disease (GBD) is an international collaboration that has been estimating the burden of diseases, injuries, and risk factors since 1990. The GBD has categorized ICD-10 codes into clinical entities, which were downloaded from the GHDx browser (<http://ghdx.healthdata.org/record/global-burden-disease-study-2016-gbd-2016-causes-death-and-nonfatal-causes-mapped-icd-codes>, last accessed 12^th^ June 2018). Causes of death and non-fatal categories were merged, and resulted in 333 categories of disease and injury. The GDB categorization forms a hierarchy, which was manually extracted from the supplementary material of the latest GBD article^1^. Further, we manually curated each category and assigned it to a physical or anatomical classification following the ICD-10 chapters and indicated whether the category was sex specific or not (Supplementary Data 4). The curation process was validated by a trained clinician. For the analysis, we only included the lowest level terms. For instance, “Non-melanoma skin cancer” has two sub-terms: ”Non-melanoma skin cancer (squamous-cell carcinoma)” and ”Non-melanoma skin cancer (basal-cell carcinoma)”. Thus, “Non-melanoma skin cancer” was not included in the analysis. In comparison, “Cervical cancer” has no sub-terms. Categories with no assigned ICD-10 codes were excluded. The final number of categories investigated, prior to any filtering based on prevalence, was 227.

### Global Burden of Disease Incidence Rates

We estimated the incidence rate of 189 GBD categories assigned to more than 100 men or women using the same model detailed for the ICD-10 terminology, described in the Methods section. We found that 136 GBD categories had a different Age adjusted Incidence Rate in men or women (AIR). There were 85 GBD categories with an increased AIR in men, and 51 in women, respectively (see Supplementary Data 5 for all 95% Bayesian Credible Intervals). The GBD categories with a large difference in AIR included breast cancer, anorexia nervosa, and alcoholic cardiomyopathy. Differences in the AIR spread across multiple functional system (Supplementary Fig. 1A). Men had, on average, a higher AIR in areas such as diseases of the respiratory system (ch10), diseases of the circulatory system (ch9), diseases of the genitourinary system (ch14), neoplasms (ch2), and perinatal disorders (ch16). Women had a higher AIR in areas such as endocrine disorders (ch4) and eye disorders (ch7).

### Global Burden of Disease Age at First Diagnosis

We studied the age at first diagnosis across the 189 GBD categories. We found that 152 categories were diagnosed at a different age for men and women (Welch’s t-test, FDR < 0.05) (see Supplementary Data 6 for mean values and 95% Confidence Intervals (CI)). In 116 out of the 152 categories, women were assigned the diagnosis at a later age. The largest weighted differences were seen with categories in the congenital malformations (ch17), diseases of the blood (ch3), and infections (ch1) (Supplementary Fig. 1B+C). Some of the largest differences included myocarditis, asthma, and conduct disorders that all had a mean difference in age of first diagnosis of approximately ten years.

### Global Burden of Disease Co-occurrence

We observed 10,957 co-occurring pairs with more than 100 occurrences in men or women. Of those, 9,990 were removed in the pre-screening. Furthermore, two pairs were excluded due to completely overlapping ICD-10 diagnoses. Hence, 967 pairs remained, of which 14 were male specific, and 56 were female specific. The procedure for estimating the relative risk and directionality was the same as the one described in the Methods section. We found a total of 486 directional pairs when calculating the sex-adjusted relative risk (defined as an elevate risk and preferred statistical direction) (see Supplementary File S7 for estimates and 95% BCI of relative risks and directionality). There were 147 directional pairs (75 in men, 72 in women) not common to men and women.

When taking sex into account there were 408 directional pairs in men, and 441 directional pairs in women, respectively. 304 directional pairs where shared between men and women. Of the 104 directional pairs unique to men, 9 included a sex-specific diagnosis. Likewise, of the 137 directional pairs unique to women 37 included a sex-specific diagnosis. Examining the strength of the directionality of the 304 directional pairs, we found that the variance was not equal and that the variance for women was higher (F=0.52, 95% CI 0.42-0.66, F-test).

For the distribution across functional groups (Supplementary Figure 3), we found that there were some incongruities between the functional groups. For instance, in women the preferred statistical direction was a diagnosis related to the infections (GBD ch1) followed by a diagnosis related to the mental disorders (GBD ch5). In men there was no pattern. Likewise, regarding the chapters neoplasms (GBD ch2) and digestive system (GBD ch11), the preferred statistical direction for men was neoplasms and then digestive system, while it was reversed for women. There were 70 directional pairs in which the relative risk was higher in men or women. In 48 cases the RR was higher in women, and in 22 cases the RR was higher in men, respectively.

We found 93 directional pairs which had a significant difference in the time between the two diagnosis for men and women (FDR <= 0.05, Mann-Whitney U test). 77 of the directional pairs had an increased time between the first and second diagnosis in women.

Using the 487, 409, and 422 directional pairs for sex-adjusted, men, and women, respectively, we identified 674, 421, and 364 linear diagnosis trajectories, respectively.

# Supplementary Note 2

## Dagger-asterisk co-occurrences

The dagger asterisk is a dual coding system first introduced in ICD version 10. It consists of two ICD-10 codes, in which the asterisk (*) indicates the manifestation or symptoms, and the dagger (†) indicates the etiology. For example, dementia in Alzheimer disease (F00*) is a symptom and Alzheimer disease with early onset (G30†) is the etiology. In the initial filtering described in the Methods section, we removed 213 dagger-asterisk pairs. We estimated the relative risk and statistical directionality separately for these. Of the 213 pairs, we found that 140 pairs passed the initial pre-filter. Of the remaining 140, we found 81 directional pairs, adjusted for sex, 72 directional pairs in men, and 70 directional pairs in women. For the sex-adjusted directional pairs, a dagger code was diagnosed before an asterisk code 74/81 times, while it for men was 66/72 times, and for women 66/70 times, respectively. Hence, our model was in good agreement with the clinical practice.

# Supplementary Note 3

## Case stories

### Respiratory disorders

In an earlier study, we found evidence of under diagnosis in chronic obstructive pulmonary disease (COPD, ICD-10:J44) using the population wide approach^2^. The present study focuses on sex specific differences and it adds further information on both diagnosis and outcome in COPD. The average age at diagnosis (67.9 years in men, 68.7 years in women, 95% CI 0.66-0.89 years, FDR < 0.05 ) is later than that seen using questionnaire methods (52-60 years of age)^3^.

Studies using case finding by pulmonary function in a primary care setting have suggested a male predominance^3–5^. However, an important earlier report warned of increasing hospitalizations for COPD in women, as well as both a higher incidence of COPD in female nonsmokers and an increasing mortality women with COPD^6^ Our findings incorporating the entire population of Denmark confirm these earlier data; the incidence of COPD is in fact the same among men and women. Furthermore, the disease trajectories add to our understanding of disease progression patterns. In this regard, once diagnosed with COPD, men are 42% more likely to receive a subsequent diagnosis of bronchiectasis (ICD-10:J47) compared to women. In contrast, women are 60% more likely to receive a subsequent diagnosis of emphysema (ICD-10:J43). Finally, as suggested in earlier studies, respiratory failure (ICD-10:J96) following a COPD diagnosis is disproportionately female; women are 50% more likely than men to suffer respiratory failure with a COPD diagnosis^6^. The increased predisposition to respiratory failure in females following a pulmonary diagnosis is not unique to COPD. While a prior multicenter study identified age > 30 but not sex as important in asthma (ICD-10:J45) treatment failures^7^, our data reveal that when using respiratory failure as an endpoint, females with asthma had a 60% higher likelihood of developing respiratory failure than males with asthma. There have been a number of potential mechanisms proposed for this progression pattern, from mechanical, differences in responses to, hormonal/cytokine differences, and issues of bias in diagnosis and care^8–12^.

### Sarcoidosis

Our study also demonstrated remarkable power in discerning sex dependent differences in sarcoidosis (ICD-10:D86), a multisystem diseases of high prevalence in Scandinavia. We found that there was a 2.5 year difference in age at first diagnosis (95% CI 2-3, FDR < 0.05, Welch’s t-test), which was consistent with a previous study that found a 2 year gap^13^. The registry based data from Denmark reveals a male predominance in the incidence (median AIR 0.2, 95% BCI 0.16-0.25). These findings contrast an earlier study in the period 1950-1982 from Sweden, Norway, Denmark, and Finland that showed a higher incidence in women^14^. Nonetheless, the authors noted a much greater incidence in Sweden compared to other Scandinavian countries as well as no sex difference in diagnosis below age 45.

### Environmental disorders

The use of health registry data allowed us to examine, at the population level, sex differences in diseases resulting from environmental exposures, such as occupational diseases. Thus, diseases such as asbestos related lung disease (GBD:B3.2.2) are strongly male biased, reflecting the male predominance in jobs in shipyards, pipe fitting, industrial insulation, and asbestos cent manufacture. The male to female relative difference in incidence of 1.88 is of similar magnitude to the female to male difference seen with breast cancer (ICD-10:C50) (1.97, 95% BCI 1.96-1.97). In contrast, the data also shows a much closer alignment in the case of malignant mesothelioma (ICD-10:C45), which results from both primary and secondary exposures^15^. Here the lesser male to female ratio of 1.21 (95% BCI 1.12-1.29) is of similar magnitude to the female to male ratio for lupus erythematosus (ICD-10:L93) (1.24, 95% BCI 1.17-1.31).

Supplementary tables 1-4

Supplementary figures 1-6

**Tables**

Supplementary Table 1: Distribution of chronic and acute diagnosis across the ICD-10 pairs with an increased relative risk and directionality.

|  | Sex-adjusted | Men | Women |
| --- | --- | --- | --- |
| Acute 🡪 Acute | 4,098 | 3,207 | 3,215 |
| Chronic 🡪 Chronic | 2,356 | 1,894 | 2,018 |
| Acute 🡪 Chronic | 2,547 | 1,972 | 2,011 |
| Chronic 🡪 Acute | 3,122 | 2,475 | 2,657 |

**Supplementary Table 2**: Overrepresentation of ICD-10 chapter combinations in diagnosis co-occurrence. An odds ratio greater than 1 indicates an overrepresentation in men.

| Chapter | Chapter name | Chapter | Chapter name | Odds Ratio | False Discovery Rate |
| --- | --- | --- | --- | --- | --- |
| 2 | Neoplasms | 11 | Digestive system diseases | 3.40 | 2.9E-04 |
| 19 | Injuries | 19 | Injuries | 1.65 | 0.035 |
| 10 | Respiratory diseases | 18 | Signs & Symptoms | 0.32 | 8.36E-05 |
| 13 | Musculoskeletal diseases | 18 | Signs & Symptoms | 0.22 | 7.29E-06 |
| 1 | Infectious diseases | 13 | Musculoskeletal diseases | 0.096 | 0.0063 |
| 2 | Neoplasms | 9 | Circulatory system diseases | 0.066 | 0.045 |
| 9 | Circulatory system diseases | 13 | Musculoskeletal diseases | 0.048 | 0.0010 |

Supplementary Table 3: Overrepresentation of end ICD-10 chapter in directional pairs with a difference in relative risk between, comparing men to women. An odds ratio greater than 1 indicates an overrepresentation in men.

| End Chapter | Chapter name | OR | FDR |
| --- | --- | --- | --- |
| 4 | Endocrine and metabolic disorders | 2.43 | 0.022 |
| 7 | Eye and Adnexa diseases | 5.12 | 0.028 |
| 12 | Skin diseases | 5.33 | 0.00097 |
| 13 | Musculoskeletal diseases | 2.20 | 0.028 |
| 10 | Respiratory diseases | 0.15 | 1.85E-07 |
| 14 | Genitourinary system diseases | 0.40 | 0.019 |

Supplementary Table 4: Overrepresentation of end ICD-10 chapter in directional pairs with a difference in time between diagnoses, comparing men to women. An odds ratio greater than 1 indicates an overrepresentation in men.

| End Chapter | Chapter name | OR | FDR |
| --- | --- | --- | --- |
| 9 | Circulatory system diseases | 3.57 | 5.43E-06 |
| 19 | Injuries | 3.58 | 2.45E-08 |
| 2 | Neoplasms | 0.27 | 0.027 |
| 10 | Respiratory diseases | 0.42 | 0.017 |

**Figures**

**
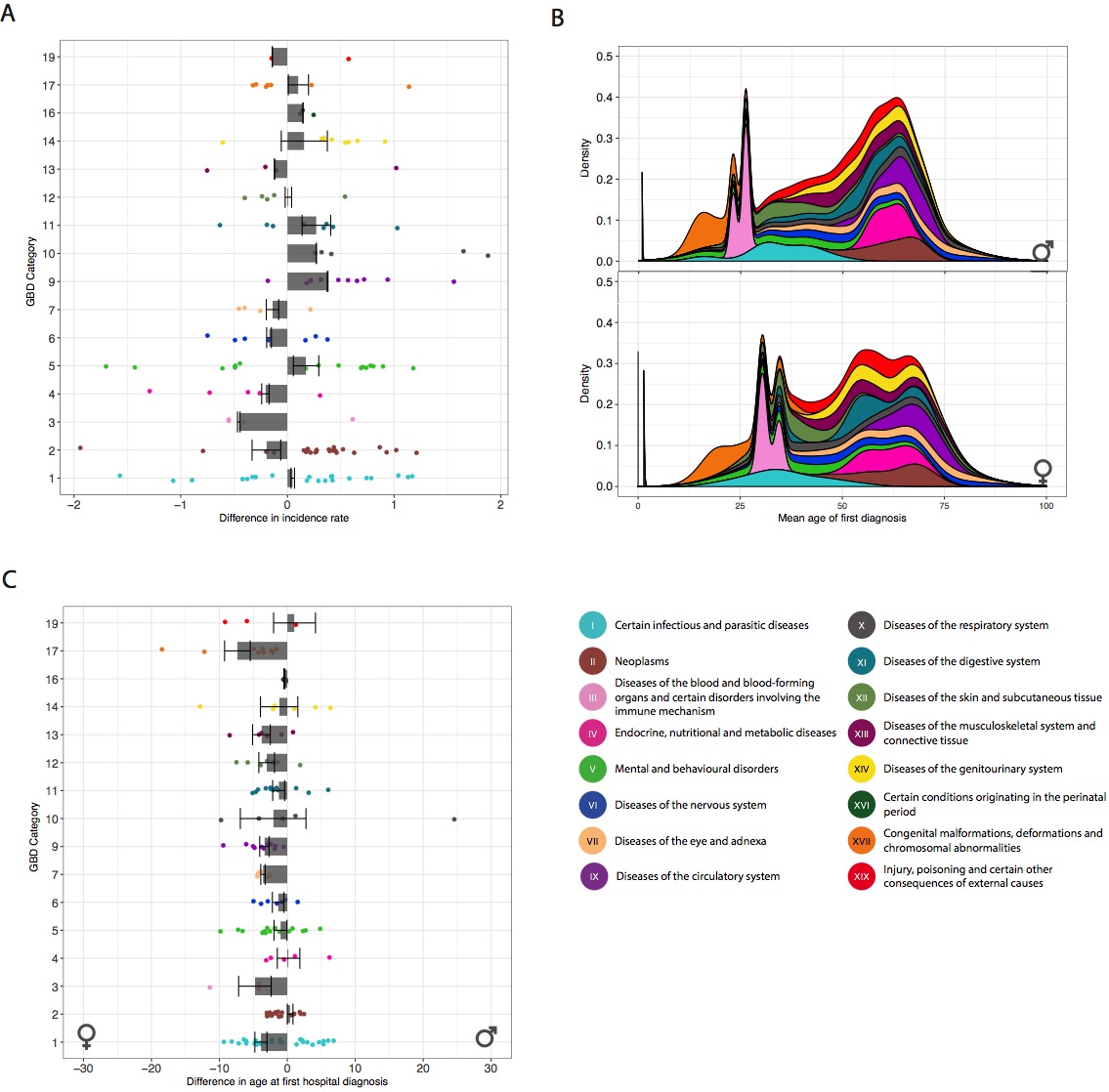
**

Supplementary Figure 1: Incidence and age at first hospital diagnosis across 189 GBD categories. (A) 85 and 51 diagnoses were found to have a higher age-adjusted incidence rate in men and women, respectively. (B) Mean age at first diagnosis for each of the 189 GBD categories studied. (C) Mean of the difference in age at first diagnosis. We found 152 diagnoses in which the age at first diagnosis was statistically significant when comparing men and women (Welch’s t-test, FDR < 0.05). Error bars are the standard error of the mean per ICD-10 chapter.

**Supplementary Figure 2:** Number of men and women diagnosed with one of the 4,155 directional pairs only found in men or women.

Supplementary Figure 3: Distribution of difference in directional strength, when compared to the joint estimate for Global Burden of Disease (GBD) and ICD-10. The distribution for women has a higher variance, and is shifted towards higher values. This indicates the directional strength is generally weaker in women.

Supplementary Figure 4: Temporal diagnosis co-occurrence across GBD categories. The distribution of 95 and 100 temporal diagnosis co-occurrences across GBD in men and women, respectively (non-sex specific diagnoses). The color scale indicates the percentage of the pairs that has the temporal directionality from the horizontal chapter to the vertical chapter. Numbers in the boxes indicate the breakdown of the overall co-occurrence figures.

**Supplementary Figure 5: (A)** For 843 temporal co-occurrences either men or women were at a higher risk. The temporal co-occurrences are colored according to the event, i.e. the diagnosis following the exposure. **(B)** Median difference in time between to diagnoses, comparing men to women. A total of 1,135 temporal comorbidities was found. The color scale indicates the percentage of the pairs that has the temporal directionality from the horizontal chapter to the vertical chapter.

Supplementary Figure 6: (A) We found 843 temporal co-occurrences where either men or women were at higher risk. The temporal co-occurrences are colored according to the event, i.e. the diagnosis following the exposure. (B) Median difference in time between to diagnoses, comparing men to women. A total of 1,135 temporal comorbidities was found.

Supplementary Figure 7: The 250 linear diagnosis trajectories based on ten directional pairs with extreme differences in relative risk illustrated as one network. The 250 trajectories were constructed from the ten directional pairs with the highest difference in relative risk between men and women. The orange edges between nodes indicate co-occurrences where the RR was elevated in women. Likewise, the green edges between nodes indicate co-occurrences where the RR was elevated in men. Two themes relating to cancer and substance abuse were apparent.

# Supplementary References

1 Vos T, Abajobir AA, Abate KH, *et al.* Global, regional, and national incidence, prevalence, and years lived with disability for 328 diseases and injuries for 195 countries, 1990-2016: a systematic analysis for the Global Burden of Disease Study 2016. *Lancet (London, England)* 2017; **390**: 1211–59.

2 Jensen AB, Moseley PL, Oprea TI, *et al.* Temporal disease trajectories condensed from population-wide registry data covering 6.2 million patients. *Nat Commun* 2014; **5**: 4022.

3 Buist AS, McBurnie MA, Vollmer WM, *et al.* International variation in the prevalence of COPD (the BOLD Study): a population-based prevalence study. *Lancet (London, England)* 2007; **370**: 741–50.

4 Miller MR, Haroon S, Jordan RE, *et al.* Clinical characteristics of patients newly diagnosed with COPD by the fixed ratio and lower limit of normal criteria: a cross-sectional analysis of the TargetCOPD trial. *Int J Chron Obstruct Pulmon Dis* 2018; **Volume 13**: 1979–86.

5 Pleasants RA, Heidari K, Wheaton AG, *et al.* Targeting Persons With or At High Risk for Chronic Obstructive Pulmonary Disease by State-based Surveillance. *COPD-JOURNAL CHRONIC Obstr Pulm Dis* 2015; **12**: 680–9.

6 Han MK, Postma D, Mannino DM, *et al.* Gender and Chronic Obstructive Pulmonary Disease. *Am J Respir Crit Care Med* 2007; **176**: 1179–84.

7 Dunn RM, Lehman E, Chinchilli VM, *et al.* Impact of Age and Sex on Response to Asthma Therapy. *Am J Respir Crit Care Med* 2015; **192**: 551–8.

8 Camp PG, Goring SM. Gender and the Diagnosis, Management, and Surveillance of Chronic Obstructive Pulmonary Disease. *Proc Am Thorac Soc* 2007; **4**: 686–91.

9 Girón RM, de Gracia Roldán J, Olveira C, *et al.* Sex bias in diagnostic delay in bronchiectasis: An analysis of the Spanish Historical Registry of Bronchiectasis. *Chron Respir Dis* 2017; **14**: 360–9.

10 Nicolson TJ, Mellor HR, Roberts RRA. Gender differences in drug toxicity. *Trends Pharmacol Sci* 2010; **31**: 108–14.

11 Kanner RE, Connett JE, Altose MD, *et al.* Gender difference in airway hyperresponsiveness in smokers with mild COPD. The Lung Health Study. *Am J Respir Crit Care Med* 1994; **150**: 956–61.

12 Becklake MR, Kauffmann F. Gender differences in airway behaviour over the human life span. *Thorax* 1999; **54**: 1119–38.

13 Judson MA, Boan AD, Lackland DT. The clinical course of sarcoidosis: presentation, diagnosis, and treatment in a large white and black cohort in the United States. *Sarcoidosis, Vasc Diffus lung Dis Off J WASOG* 2012; **29**: 119–27.

14 Milman N, Selroos O. Pulmonary sarcoidosis in the Nordic countries 1950-1982. Epidemiology and clinical picture. *Sarcoidosis* 1990; **7**: 50–7.

15 Panou V, Vyberg M, Meristoudis C, *et al.* Malignant mesothelioma in 91 danish women: The environmental asbestos exposure. *J Clin Oncol* 2017; **35**: 8560.
