# Supplementary material for: Population-wide analysis of differences in disease progression patterns in men and women
Source: Nat Commun. 2019 Feb 8;10:666. doi: 10.1038/s41467-019-08475-9 (PMC6368599; doi:10.1038/s41467-019-08475-9)
Supplement: Supplementary file 3 — Description of Additional Supplementary Files [file 41467_2019_8475_MOESM3_ESM.pdf]

Description of Additional Supplementary Files

File Name: Supplementary Data 1
Description: ICD-10 diagnosis incidence and differences for men and woman, and the 95% Bayesian Credible Interval.

File Name: Supplementary Data 2
Description: ICD-10 age of first hospital diagnosis for men and women, the mean difference, and the 95% Confidence Interval and the False Discovery Rate.

File Name: Supplementary Data 3
Description: ICD-10 diagnosis co-occurrences and directionality, and the 95% Bayesian Credible Interval.

File Name: Supplementary Data 4
Description: ICD-10 and Global Burden of Disease mapping and sex-specificity.

File Name: Supplementary Data 5
Description: Global Burden of Disease diagnosis incidence and differences for men and woman, and the 95% Bayesian Credible Interval.

File Name: Supplementary Data 6
Description: Global Burden of Disease age of first hospital diagnosis for men and women, the mean difference, and the 95% Confidence Interval and the False Discovery Rate.

File Name: Supplementary Data 7
Description: Global Burden of Disease diagnosis co-occurrences and directionality, and the 95% Bayesian Credible Interval.

File Name: Supplementary Data 8
Description: ICD-10 codes and sex specificity.
